# Supplementary material for: Fast-Rising Electric Pulses by Reducing Membrane Tension for Efficient Membrane Electroporation
Source: Membranes (Basel). 2025 May 16;15(5):151. doi: 10.3390/membranes15050151 (PMC12113182; doi:10.3390/membranes15050151)
Supplement: Supplementary file 1 [file membranes-15-00151-s001.zip › membranes-3539535-supplementary.pdf]

membranes

MDPI

Supplementary Materials

Fast-Rising Electric Pulses by Reducing Membrane Tension for Efficient Membrane Electroporation

Ping Ye, Lulu Huang and Kuiwen Zhao *

School of Health Science and Engineering, University of Shanghai for Science and Technology, Shanghai 200093, China; iamyeping@usst.edu.cn (P.Y.); 223332599@st.usst.edu.cn (L.H.)
* Correspondence: kuiwen@arcticous.com; Tel.: +86-13564896728

Table S1. The electric field schemes for the rise phase and the plateau phase.

Rise time (ns)	MD Time step (ps)	Peak electric field (V/nm)	Constant time (ns)	Peak electric field (V/nm)	Constant time (ns)	Peak electric field (V/nm)	Constant time (ns)
1	0.02	0.16	50	0.18	10	0.20	10
5	0.02	0.16	50	0.18	10	0.20	10
10	0.02	0.16	50	0.18	10	0.20	10
15	0.02	0.16	50	0.18	10	0.20	10
20	0.02	0.16	50	0.18	10	0.20	10
30	0.02	0.16	50	0.18	10	0.20	10

Table S2. The average time of pore formation measured from the onset of the peak electric field.

Rise time (ns)	Peak electric field (V/nm)	Pore time (ns)	Peak electric field (V/nm)	Pore time (ns)
1	0.16	28.3 ± 1.7	0.18	8.4 ± 0.2
5	0.16	32.4 ± 1.1	0.18	4.9 ± 0.5
10	0.16	33.9 ± 0.6	0.18	8.1 ± 0.3
15	0.16	33.3 ± 1.8	0.18	4.6 ± 0.3
20	0.16	32.8 ± 1.4	0.18	2.8 ± 0.2
30	0.16	28.9 ± 2.4	0.18	0.8 ± 0.1

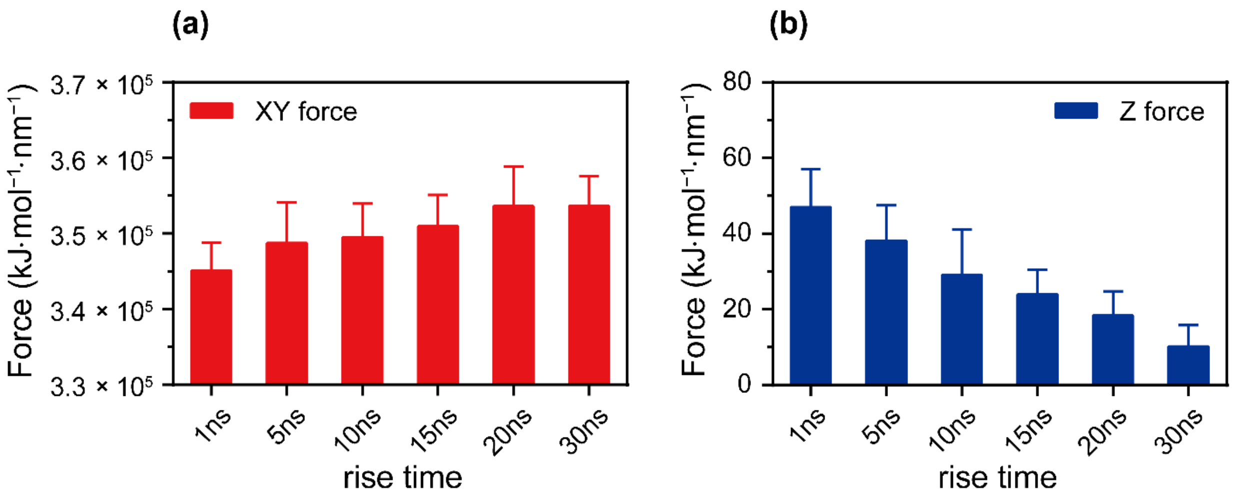

Figure S1. Coulomb force acted on the phospholipid membrane by an electric field with various electric field rise time (peak electric field: 0.18 V/nm). (**a**) The Coulomb force acting on the phospholipid membrane in the XY plane under different electric field rise time. This Coulomb force was calculated by vector summation of the X and Y directions. (**b**) The Coulomb force acting on the phospholipid membrane in the Z direction under different electric field rise time. The error bars represent the standard deviation of the data within a 1 ns time window (excluding the electric field rise time phase) centered around the pore formation moment.

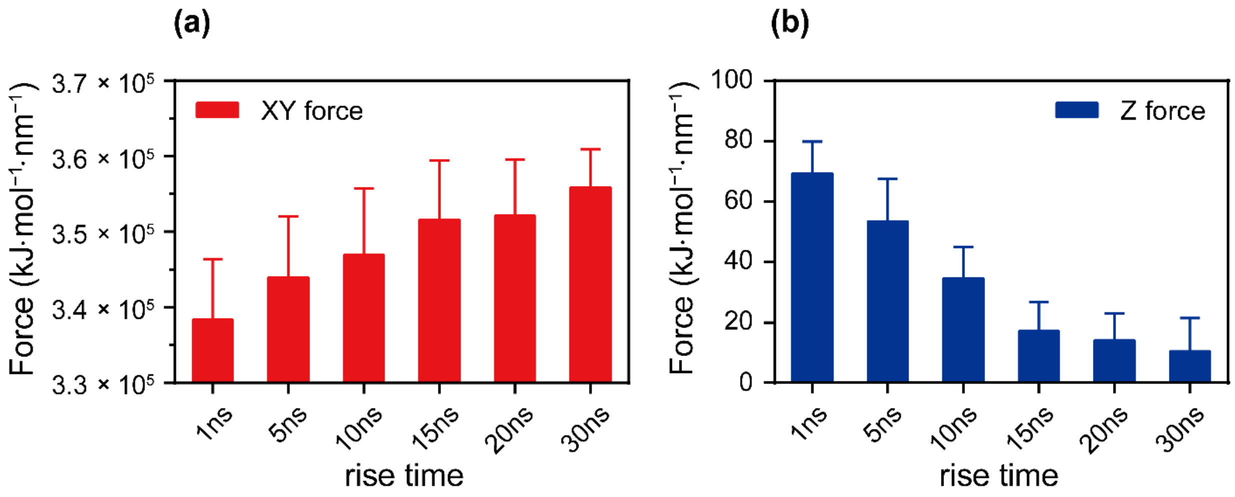

Figure S2. Coulomb force acted on the phospholipid membrane by an electric field with various electric field rise time (peak electric field: 0.20 V/nm). (**a**) The Coulomb force acting on the phospholipid membrane in the XY plane under different electric field rise time. This Coulomb force was calculated by vector summation of the X and Y directions. (**b**) The Coulomb force acting on the phospholipid membrane in the Z direction under different electric field rise time. The error bars represent the standard deviation of the data within a 1 ns time window (excluding the electric field rise time phase) centered around the pore formation moment.
